# Induction of IL-6 and CCL5 (RANTES) in human respiratory epithelial (A549) cells by clinical isolates of respiratory syncytial virus is strain specific

**DOI:** 10.1186/1743-422X-9-190

**Published:** 2012-09-10

**Authors:** Ruth Levitz, Rachel Wattier, Pamela Phillips, Alexandra Solomon, Jessica Lawler, Isaac Lazar, Carla Weibel, Jeffrey S Kahn

**Affiliations:** 1 Department of Pediatrics, University of Texas Southwestern Medical Center, Dallas, TX, USA; 2 Department of Microbiology, University of Texas Southwestern Medical Center, Dallas, TX, USA; 3 Yale University School of Medicine, New Haven, CT, USA; 4 The University of California San Francisco, San Francisco, CA, USA; 5 Ben Gurion University, Beer Sheva, Israel

**Keywords:** Respiratory syncytial virus, Clinical isolates, IL-6, RANTES, CCL5, Respiratory syncytial virus genome

## Abstract

**Background:**

Respiratory syncytial virus (RSV) is the major respiratory pathogen of infants and young children. During each seasonal epidemic, multiple strains of both subgroup A and B viruses circulate in the community. Like other RNA viruses, RSV genome replication is prone to errors that results in a heterogeneous population of viral strains some of which may possess differences in virulence. We sought to determine whether clinical isolates of RSV differ in their capacity to induce inflammatory cytokines IL-6 and CCL5 (previously known as RANTES [regulated upon activation, normal T-cell expressed and secreted protein]), which are known to be induced in vitro and in vivo in response to RSV, during infection of A549 cells.

**Results:**

Screening of subgroup A and B isolates revealed heterogeneity among strains to induce IL-6 and CCL5. We chose two subgroup B strains, New Haven (NH)1067 and NH1125, for further analysis because of their marked differences in cytokine inducing properties and because subgroup B strains, in general, are less genetically heterogeneous as compared to subgroup A strains. At 12 and 24 hours post infection RSV strains, NH1067 and NH1125 differed in their capacity to induce IL-6 by an order of magnitude or more. The concentrations of IL-6 and CCL5 were dependent on the dose of infectious virus and the concentration of these cytokines induced by NH1125 was greater than that of those induced by NH1067 when the multiplicity of infection of NH1067 used was as much as 10-fold higher than that of NH1125. The induction of IL-6 was dependent on viable virus as infection with UV-inactivated virus did not induce IL-6. The difference in IL-6 induction most likely could not be explained by differences in viral replication kinetics. The intracellular level of RSV RNA, as determined by quantitative RT-PCR, was indistinguishable between the 2 strains though the titer of progeny virus produced by NH1125 was greater than that produced by NH1067 at 16, 24 and 36 hours but essentially equal at 48 and 72 hours. Full genome sequencing of the 2 strains revealed 193 polymorphisms and 4 insertions in NH1067when compared to NH1125 (2 single base insertions in non-coding regions and 2 duplications of 3 and 60 bases in the RSV G gene). Of the polymorphisms, 147 occurred in coding regions and only 30 resulted in amino acid changes in 7 of the RSV genes.

**Conclusions:**

These data suggest that RSV strains may not be homogeneous with regard to pathogenesis or virulence. Identification of the genetic polymorphisms associated with variations in cytokine induction may lead to insights into RSV disease and to the development of effective antiviral agents and vaccines.

## Background

Respiratory syncytial virus (RSV) is a major respiratory pathogen of infants and young children as well as the elderly and immunocompromised populations [1]. Clinical isolates can be categorized into 1 of 2 major subgroups of RSV, A or B, by immunological or genetic methods [2,3]. Within each subgroup, there are several distinct genotypes, a variety of which co-circulate during yearly epidemics [3-5].

The inflammatory response to RSV infection plays a major role in the disease pathogenesis and this inflammation may result in signs of restrictive and obstructive lung disease long after RSV can be detected in respiratory secretions. RSV elicits the production of a variety of cytokines and chemokines during and after infection [6,7]. Both in vivo and in vitro data suggest that IL-6, a pro-inflammatory cytokine, is a key component of the response of the host to RSV infection. Microarray analysis of RSV-infected human alveolar type II epithelial (A549) cells reveals that IL-6 mRNA was induced ~20-fold 30 minutes after infection [8]. IL-6 is present in the respiratory secretions of RSV-infected individuals and serum levels of IL-6 are increased during RSV infection [9,10]. The mechanism by which RSV induces IL-6 production is poorly understood. The chemokine CCL5 (previously known as RANTES [regulated upon activation, normal T-cell expressed and secreted protein]) is induced in humans and animal models of RSV and evidence suggests that this molecule plays an important role in pathogenesis [6,11]. Therefore, exploration of the induction of IL-6 and CCL5 by clinical isolates of RSV may be insightful.

Several groups, including our own, have demonstrated a potential association between viral genotype and severity of illness, suggesting that clinical isolates of RSV may differ in virulence [9,12-15]. However, the study of strain-specific factors in vivo is complicated by a variety of host factors influencing the severity of infection. In order to eliminate these factors, we chose to study strain differences in a cell culture model system. As such, we screened and plaque purified clinical isolates of RSV and assessed their ability to induce cytokines in human alveolar type II epithelial (A549) cells. The results demonstrate that clinical isolates differ dramatically in their ability to induce certain cytokines in cell culture. These findings suggest that the genetic variability of clinical isolates of RSV may contribute to the severity of infection caused by the virus.

## Results

### Screening of clinical isolates of RSV for their ability to induce IL-6

**Figure 1:**
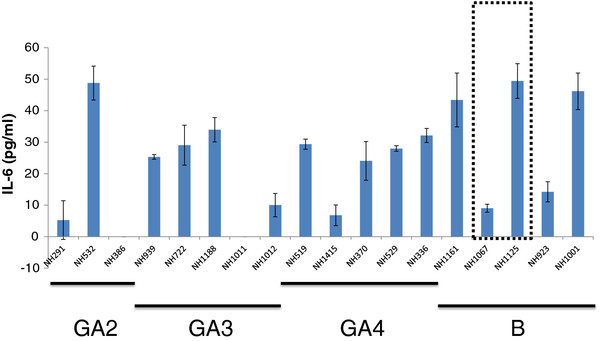
**Screening of clinical isolates of RSV for induction of IL-6.** A549 cells were infected with multiplicity of infection (moi) 1 and IL-6 concentrations in cell culture supernatants at 24 hours post infection were measured by ELISA. Subgroup **A** isolates (clades GA2, GA3 and GA4) and subgroup B isolates are designated by the bars below the graph. Subgroup **B** isolates NH1067 and NH1125, chosen for subsequent experiments, are boxed.

**Figure 2:**
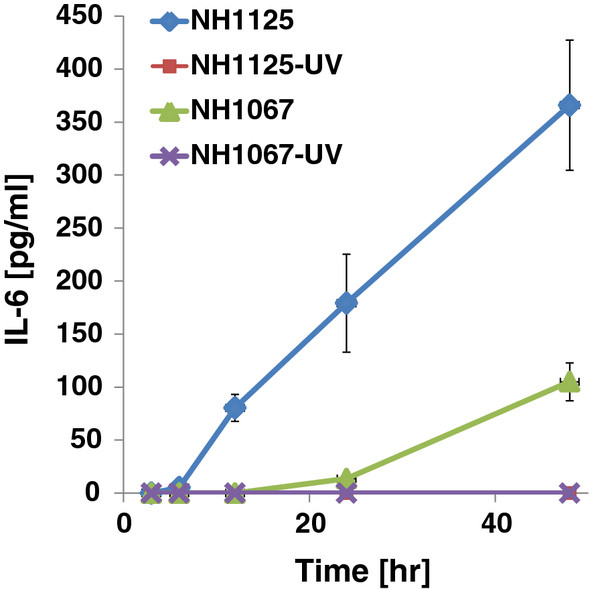
**Induction of IL-6 by clinical isolates NH1067 and NH1125.** A549 cells were infected with multiplicity of infection (moi) 1 and IL-6 concentrations in cell culture supernatants were measured by ELISA. Ultraviolet light (UV) inactivated virus was used as a control. Standard error bars are displayed.

Since 1998, we have propagated clinical strains of RSV in cell culture and have performed phylogenetic analysis of >200 of these isolates (data not shown). From this collection of clinical isolates, we screened both subgroup A and B isolates for their ability to induce the secretion of IL-6 during infection of the pulmonary epithelial cell line A549. We chose genetically diverse strains of subgroup A (representing at least 3 distinct clades [12]) and B isolates (based on previous phylogenetic analysis (data not shown). The strains screened were not homogenous in their cytokine induction properties (Figure 1). To further explore this phenomenon, we chose 2 subgroup B strains, New Haven (NH)1067 and NH1125 for further investigation. These strains were chosen for 2 reasons: 1) there were marked differences observed in the levels of IL-6 induced between these strains (Figure 1, boxed) and; 2) subgroup B isolates are less genetically heterogeneous as compared to subgroup A isolates. Our hypothesis was that there would be fewer polymorphisms between pairs of isolates of subgroup B as compared to subgroup A. If so, study of subgroup B isolates would be preferable in eventually identifying the viral genetic markers responsible for cytokine induction. Both strains were isolated from respiratory specimens collected in 2002. At 12 and 24 hours post-infection, the level of IL-6 induced by NH1125 was at least an order of magnitude greater than that by NH1067 (Figure 2). The mechanism of this induction was explored. UV-inactivated virus (both strains) failed to induce IL-6 (Figure 2) suggesting that viable virus, or at least virus with transcriptional and genome replication activity was required for induction of IL-6. These findings also suggest that virus binding to the target cell, which presumably still occurs with UV-inactivated virus, was not sufficient for IL-6 induction.

### Dynamics of replication of clinical isolates NH1067 and NH1125

**Figure 3:**
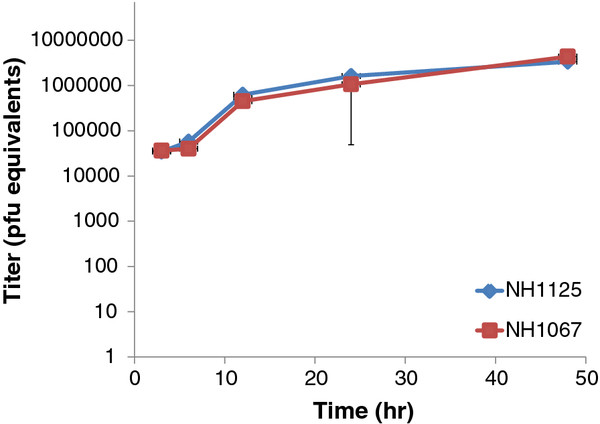
**Viral replication kinetics of NH1067 and NH1125.** Intracellular viral genomic RNA levels were determined by real time reverse transcriptase PCR. RNA quantity is displayed as pfu equivalents based on a standard curve (data not shown).

**Figure 4:**
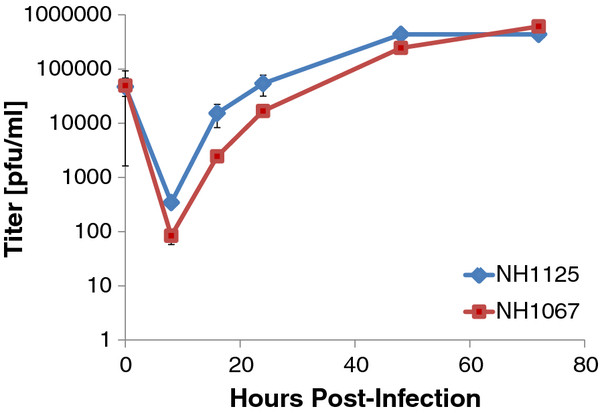
**Plaque assay replication curves of NH1067 and NH1125.** A549 cells were infected with a moi of 0.2. At specific times after infection, cells were harvested and viral titer was determined by plaque assay.

One potential explanation for the observed differences in cytokine induction by NH1067 and NH1125 was that the replication kinetics of the 2 viruses differed. To address this possibility, viral replication kinetics were assayed by 2 means: quantitative real time PCR and traditional viral growth assays. Quantitative RT-PCR, using primers specific for the negative sense genome strand, revealed that the kinetics of genome replication of NH1125 and NH1067 were identical (Figure 3). As shown in Figure 4, production of infectious virus from NH1067- or NH1125-infected cells at 48 and 72 hours post-infection are identical though at earlier times, there is less infectious virus detected in NH1067-infected cells.

### Induction of cytokines is dose-dependent

**Figure 5:**
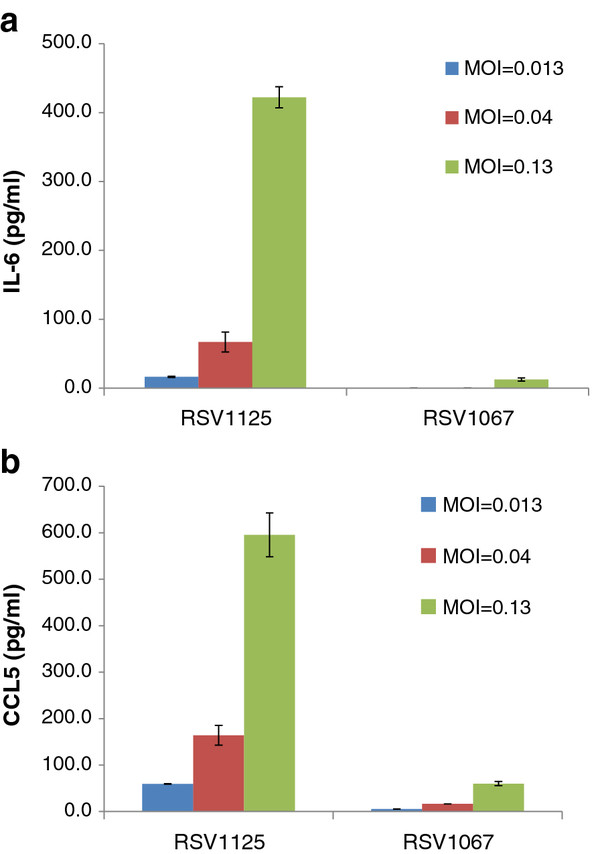
**Dose response of induction of IL-6 and CCL5 in A549 cells during infection with NH1067 and NH1125.** A549 cells were infected with a moi of 0.013, 0.04 or 0.13 with either NH1067 or NH1125. At 24 hours post infection, supernatant concentrations of IL-6 (**a**)and CCL5 (**b**) were measured. Standard error bars are displayed.

To further explore the differences in cytokine induction by strains NH1067 and NH1125, infectious dose–response experiments were performed. A549 cells were infected with a multiplicity of infection (moi) of 0.013, 0.04 or 0.13 and supernatant levels of IL-6 and CCL5 were determined at 24 hours post-infection. As shown in Figure 5 (a and b), induction of IL-6 and CCL5 by NH1125 was dependent on the infectious dose of virus. There also appeared to be a dose-dependent induction of CCL5 in cells infected with NH1067. However, NH1067 was a comparatively very poor inducer of both cytokines. Of note, the levels of IL-6 and CCL5 induced by NH1067 at a moi of 0.13 was essentially equivalent to the levels of IL-6 and CCL5 induced by NH1125 at a moi of 0.013, a ten-fold difference in infectious dose.

### Genome sequence comparisons of NH1067 and NH1125

The genome of NH1067 contained 15,283 nucleotides [GenBank: accession JQ582844] and the genome of NH1125 contained 15,216 nucleotides [GenBank: accession JQ582843]. Overall, there were 194 polymorphisms between the 2 viruses (Table 1). Nucleotide differences were observed in every open reading frame (ORF) though the majority (117/147) of the polymorphisms in the coding sequences were synonymous. The exception was the RSV G gene. Of the 23 polymorphisms detected in the RSV G gene of NH1067 and NH1125, 13 resulted in mis-sense mutations while 10 were silent. This is not surprising as the RSV G gene is known to be the most variable of all genes in the RSV genome [16,17]. The RSV G gene of NH1067 contained 2 duplications, 3 and 60 nucleotides in length, which were not present in NH1125. Viruses containing the 60 base duplication were first identified in Buenos Aires in 1999 [18]. Subsequently, RSV subgroup B viruses with this 60 base insertion have been identified worldwide [19].

**Table 1:** Sequence comparisons of clinical isolates NH1067, NH1125

**Genomic segment**^**a**^	**Length in bases**^**b**^	**Nucleotide differences**	**Non-sense**^**c**^	**Mis-sense**^**c**^	**Silent**^**c**^
3’end-NS1	98	1			
NS1 gene	420	1			1
NS1-NS2	107	0			
NS2 gene	375	4		1	3
NS2-N	136 (137)	6			
N gene	1176	16		1	15
N-P	32	0			
P gene	726	5		1	4
P-M	186 (188)	11			
M gene	771	10			10
M-SH	268	10			
SH gene	198	4			4
SH-G	189	4			
G gene	888 (951)	23		13	10
G-F	88	0			
F gene	1725	20		4	16
F-M2-1	225 (226)	8			
M2-1 gene	588	4		2	2
M2-1-M2-2^d^	35	0			
M2-2 gene	273	4			4
M2-2-L	65	2			
L gene	6501	59		8	51
L-5’end	216	2			
Total	15216 (15283)	194	0	30	120

^a^Gene coding sequences (“gene”) or intergenic (inter-ORF) segment listed unless otherwise noted (3’ and 5’ end).

^b^NH1067 length in () when it differs from NH1125. NH1067 G gene contains a 3 base and 60 base insertion.

^c^Non-sense, mis-sense and silent nucleotide differences between the 2 strains are listed for coding sequences only.

^d^M2-1 and M2-2 overlap by 35 nucleotides.

### Association of genotype and cytokine induction phenotype

**Figure 6:**
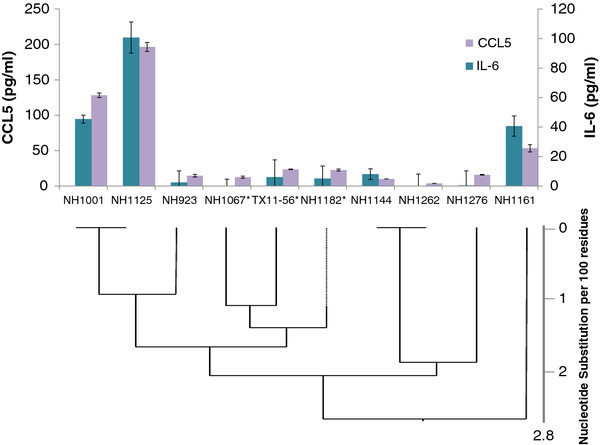
**Phenotypic and genotypic association of induction of IL-6 and CCL5 by clinical isolates of RSV.** A549 cells were infected with clinical isolates at a moi of 0.13. Cytokine levels (IL-6 and CCL5) in cell culture supernatants were measured at 16 hours after infection. Groupings of clinical isolates were based on phylogenetic analysis of the RSV G gene as demonstrated by the phylogram beneath the graph. Viruses containing the RSV G gene 60 base duplication are designated with the *.

To determine whether other subgroup B isolates had cytokine-inducing phenotypes similar to NH1067 or NH1125, 8 additional viruses were assayed for their ability to induce IL-6 and CCL5. These additional viruses were chosen because they were NH1067-like, NH1125-like or distantly related to NH1067 and NH1125 based on their G gene sequences some of which contained the 60-base duplication. As shown in Figure 6, the induction profiles correlated, for the most part, with genotype. NH1001 and NH1125 had similar cytokine induction profiles and were closely related genetically. Likewise, NH1067, Texas (TX)11-56 and NH1182, closely related viruses, were poor cytokine inducers (low inducers). However, NH1161, a relative outlier, had an IL-6 induction phenotype that was similar to NH1001. Of note, the low induction phenotype does not seem to correlate with the G gene duplication detected in NH1067. Viruses containing the 60 base duplication (NH1067, NH1182, TX11-56; designated with the * in Figure 5) and viruses lacking this duplication (NH923, NH1144, NH1262, NH1276) all had a low induction phenotype. Polymorphisms in the F gene in NH1067 and NH1125 resulted in 4 amino acid differences at positions 67, 292, 490 and 529. It is unlikely that any of these 4 mutations were the basis for the observed phenotype. Sequencing of the F gene of the viruses represented in Figure 5 did not reveal a correlation between specific amino acid residues at these positions in the F protein and cytokine induction phenotype (data not shown).

## Discussion

RSV elicits the production of a wide variety of cytokines and chemokines during and after infection [6,7,20-23]. There is now mounting evidence that the type of immune response to RSV, specifically the spectrum of host cytokine and chemokine production, ultimately affects disease pathogenesis and chronicity [6]. Cytokine expression likely affects the balance between viral clearance and progression of disease. Therefore, there is a critical need to identify viral factors that regulate cytokine expression during RSV infection [6].

The vast majority of studies into RSV pathogenesis have used laboratory strains or recombinant virus derived from these laboratory strains. These laboratory strains were isolated decades ago and the passage histories of these strains are not known or are poorly documented. Several studies suggest that sole investigation of laboratory strains may not be ideal to define and characterize RSV pathogenesis. For example, Line 19, a potential vaccine strain derived from a subgroup A isolate possesses biological features that differ from the reference strain A2 (isolated in Australia in 1961) [24]. These phenotypic differences have been mapped to polymorphisms in the RSV F gene [25]. Reference strain A2 differs from several clinical isolates in its ability to replicate on primary human bronchial epithelial cells and to induce interferon-inducible protein 10 and CCL5 suggesting that A2 alone may not be the ideal strain to study RSV pathogenesis [26]. Recently, Stokes et al. demonstrated that several clinical isolates differ in their capacity to induce airway mucous production and virulence in mice [27]. Therefore, the study of clinical isolates will likely yield important insights into the fundamental aspects of pathogenesis.

At least 2 RSV proteins have been implicated in the induction of IL-6, RSV F and G. In monocytes, RSV F stimulates IL-6 through interactions with CD14+/TLR4 complex by promoting nuclear translocation of NF-ĸB [28]. IL-6, along with other cytokines, such as IL-1β and IL-8, play an important role in neutrophil and macrophage chemotaxis. The cellular inflammatory response during severe RSV infection is characterized by a preponderance of neutrophils and macrophages, suggesting that the expression of IL-6 at the site of infection is an essential feature in disease progression [29]. Differences in IL-6 induction may explain, in part, differences in severity of illness seen with RSV infection. Indeed, clinical studies of both infants and adults have demonstrated that single nucleotide polymorphisms in the IL-6 promoter (position −174) were associated with a greater severity of illness suggesting a pivotal role of IL-6 in RSV pathogenesis [30,31]. Our data suggests that variability in the induction of IL-6 may, at least, partially explain differences in the observed severity of human RSV disease associated with infection due to specific RSV genotypes [9,12-15]. The RSV F protein of NH1067 and NH1125 differs at only 4 amino acid residues (positions 67, 292, 490 and 529). It did not appear that these amino acid changes can account for the differences in induction of IL-6 observed because the specific amino acid residues at these positions did not correlate to induction phenotype when other strains were examined.

Several lines of evidence indicate that the RSV G glycoprotein also plays a role in pathogenesis and can induce IL-6 among other cytokines [32-37]. Monocyte stimulation experiments using recombinant viruses with altered or absent G gene and synthetic peptides, corresponding to regions of the RSV G protein, revealed that portions of the highly conserved cysteine-rich region, specifically amino acids 164–176, inhibit the innate immune response [37]. The CX3C motif (amino acid 182–186) of the RSV G glycoprotein mimics the CX3C chemokine fractalkine, competes for the chemokine-specific receptor and interferes with fractalkine-mediated leukocyte chemotaxis [32]. RSV deficient in the soluble form of G (generated by initiation at the second AUG corresponding to amino acid 48) induced a pro-inflammatory response in A549 cells [38]. Amino acids 164–176 and 182–186 were identical in NH1067 and NH1125. Both strains contained a methionine at position 48. Therefore, these regions of the RSV G gene, although likely important for cytokine induction pathways, cannot account for the differences observed in IL-6 induction between NH1067 and NH1125.

Our data suggest that the differences in the RSV F protein between NH1067 and NH1125 are unlikely to account for the differences in cytokine induction phenotype strongly suggesting that other viral factors, perhaps in other domains of the G protein, may play a role in the differences in cytokine induction observed in the clinical isolates. These factors may not necessarily be viral proteins. A single nucleotide substitution in the transcriptional start signal of the M2 gene, within a non-coding region of the RSV genome [39] is the major determinant of the temperature-sensitive, attenuated phenotype of a potential vaccine candidate. However, the consensus transcriptional start and stop signals for each gene were identical between NH1067 and NH1125. It appears as though viral transcriptional and/or replication activity rather than binding of virion-associated glycoproteins to cellular receptors is required as UV-inactivated virus failed to induce IL-6, an observation that has been reported elsewhere [40,41].

RSV causes a wide-spectrum of disease and this may be due, in part, to the differences in biological properties of the infecting virus. This phenomenon has been observed with other viruses. For example, mutations in the HCV NS5A gene are associated with a sustained virological response to interferon therapy [42]. A neurotropic variant of HIV, containing a mutation in the envelope gene, has been identified, which is present at a high frequency in brain tissue in AIDS patients with dementia [43]. Mutations in several genes may account for the increased virulence of the 1918 influenza pandemic strain [44]. Identification of the viral gene(s) involved in the induction of key cytokines, chemokines and inflammatory mediators would potentially lead to targeted antiviral therapies and would be a significant advance in the study of RSV pathogenesis.

## Conclusion

This work demonstrates that clinical isolates of RSV are not homogeneous in their ability to induce specific cytokines. Screening of a relatively small number of clinical isolates resulted in the identification of strains, NH1067 and NH1125, with markedly different cytokine-inducing phenotypes. This work demonstrates the utility of investigating clinical isolates, whose naturally-occurring mutations result in distinct phenotypes. These data suggest that further investigation, particularly with the use of primary human cells (airway epithelial cells or PBMCs), of these and other clinical isolates of RSV may yield insights into pathogenesis and virulence.

## Methods

### Viruses and cells

HEp-2 (CCL-23) and A549 (CCL-185) cells were obtained from the American Type Culture Collection (Manassas, VA) and cultivated in EMEM with 10 % fetal bovine serum. HEp-2 cells were used in the initial propagation and titration of the clinical strains; A549 cells were used in the cytokine experiments described below. Clinical isolates were obtained from RSV-infected individuals as described previously from New Haven, Connecticut [12] and Dallas, Texas. Viruses used in this study were propagated from specimens submitted to the clinical (diagnostic) laboratory (in New Haven, CT and Dallas, TX). All specimens from which viruses were obtained were submitted as part of routine care. Only left over material was used for viral propagation. Collection of specimens from the Clinical Virology Laboratory at Yale-New Haven Hospital was approved by the Yale University Human Investigations Committee. The single isolate from Dallas, Texas was propagated from a de-identified clinical specimen obtained from the Clinical Microbiology Laboratory at Children’s Medical Center, Dallas. Collection and use of clinical isolates followed all institutional requirements and guidelines and was consistent with policies and regulations for the use of patient derived materials.

Isolates were plaque purified 3 times on HEp-2 cells. Working stocks were prepared using 30%-60% (w/v) non-continuous sucrose density centrifugation (SW-28, 28,000 rpm, 4°C for 90 minutes). The virus containing band at the 30%-60% interface was collected, distributed into aliquots, and stored at −80°C. Viral titers were determined using a plaque assay [45]. Plaques were detected with an immunohistochemical staining technique using either goat anti-RSV HRP-conjugated antibody (Fitzgerald Industries International, Acton, MA) or a primary anti-RSV antibody (palivizumab, MedImmune, Gaithersberg, MD) and secondary HRP conjugated anti-human antibody (Jackson ImmunoResearch Laboratories, Inc., West grove, PA). Viruses were inactivated by subjecting viral inoculums to 2 doses of 5,000 joules of ultraviolet (UV) light. UV-inactivation was confirmed by cell culture.

### Cytokine assays

Monolayers of A549 cells were infected with sucrose-purified clinical isolates of RSV at the moi designated in the text. After 90 minutes of infection, the inoculum was removed, the cells were washed with serum-free media and fresh media (F-12 Kaighn’s modification) containing 5% FBS was added to the infected monolayers. For cytokine analysis, supernatants were collected, clarified by centrifugation, snap frozen in liquid nitrogen and stored at −80°C until the specific assay was performed. For the initial screening of clinical isolates, the concentration of IL-6 was determined by ELISA using the DuoSet Human IL-6 ELISA kit (R&D Systems, Minneapolis, MN) according to the manufacturer’s specifications. Standard curves with known concentrations of IL-6 were performed for each assay. All samples were analyzed on the same ELISA plate to minimize technical bias and there were at least 3 biological replicates for each measurement. For experiments that measured concentrations of IL-6 and CCL5, BioPlex assays were performed using Bio-Plex Pro™ with conjugated magnetic beads according to the manufacturer’s instructions. Cytokine data were analyzed using Bio-Plex Manager™ version 4.1.1 software.

### Real time PCR

Total RNA was extracted from A549 cell monolayers using the RNeasy Mini kit and QIAshredder columns (QIAGEN, Valencia, CA) according to the manufacturer’s recommendations. RNA extracts were treated with RNase-free DNase I (Roche), then reverse transcribed with Moloney murine leukemia virus reverse transcriptase (New England BioLabs) with a primer specific for the genomic (negative-sense) copy of the RSV N gene, 5'-ATGGCTCTTAGCAAAGTC-3'. PCR amplification of cDNA was performed in a reaction mixture containing 5 μL cDNA template, forward (5'-CTGTCATCCAGCAAATACACTATTCA-3') and reverse (5'-GCACATCATAATTGGGAGTGTCA-3') primers at a concentration of 400 n*M* each, and 12.5 μL iQ SYBR Green Supermix (Bio-Rad) in a total volume of 25 μL. Reaction conditions were as follows: 95°C for 10 minutes, followed by 40 cycles of 95°C for 15 seconds and 60°C for 1 minute. For cDNA standards, RNA was extracted from plaque-titered stocks of the laboratory reference B strain 8/60 using the QIAamp Viral RNA Mini Kit (Qiagen), reverse-transcribed as above, and serially diluted. RSV genomic RNA quantity in each sample was calculated in “pfu equivalents.”

### Viral genome sequencing, phylogenetic analysis and real time PCR

Viral RNA was extracted from clarified supernatants or sucrose-purified virus using QIAamp® Viral RNA Mini kit (QIAGEN). Reverse transcriptase and PCR were performed as previously described [12]. Primers used for the sequencing of clinical isolates are listed in Additional file 1. For sequence of the ends of the viral genome, purified viral RNA was circularized by ligation using T4 RNA ligase (Epicentre, Madison, WI) as described elsewhere [46]. Primers were designed (see Additional File 1) to span the region of the ligated ends. The exact nucleotide at the 3’ and 5’ ends were determined by comparison to available genome sequences. DNA sequencing of PCR products was performed by the McDermott Center Sequencing Core using Applied Biosystems Inc. (ABI) Big Dye Terminator 3.1 chemistry and analyzed on ABI capillary instruments. Sequence analysis including alignment and integration of contigs, sequence comparisons and alignments, and phylogenetic analysis were performed using DNASTAR Lasegene 8 software (MegAlign and Seqman).

## Competing interests

The authors declare that they have no competing interests.

## Authors’ contributions

RL, RW, PP, JL, IL, CW, JSK identified and isolated clinical strains of RSV. RL, RW, IL performed cytokine assays. RL, PP, AS, JL, JSK participated in the sequencing and analysis of the RSV genomes. RL, RW carried out the viral replication assays. JSK conceived of the study, provided oversight for all aspects of the study and prepared the draft of the manuscript. All authors read and approved the final manuscript.

## Authors’ information

RW current institution is The University of California San Francisco. IL current institution is Ben Gurion University, Beer Sheva, Israel. AS is an undergraduate student at Cornell University. JL is an undergraduate student at Texas A&M.

## Supplementary Material

Additional file 1 Primers Used for Sequencing of the genome of RSV isolates. Click here for file
