# Supplementary material for: Induction of IL-6 and CCL5 (RANTES) in human respiratory epithelial (A549) cells by clinical isolates of respiratory syncytial virus is strain specific
Source: Virol J. 2012 Sep 10;9:190. doi: 10.1186/1743-422X-9-190 (PMC3463437; doi:10.1186/1743-422X-9-190)
Supplement: Additional file 1 — Primers Used for Sequencing of the genome of RSV isolates. [file 1743-422X-9-190-S1.doc]

Additional File 1. Primers Used for Sequencing of the genome of RSV isolates

| **Set #** | **F/R** | **Sequence** | **Virus^a^** | **Genome coordinates** |
| --- | --- | --- | --- | --- |
| **1** | F | GCG TAC TAC AAA CTT GCA CAT TCG | NH1125  NH1067 | 13-36 |
|  | R | GTG TGT GAT GAT TTC TTT GGT GAG AGA TG | NH1125  NH1067 | 708-736 |
| **2** | F | GCA TTA GCC AAA GCA GCA ATA CAT AC | NH1125  NH1067 | 216-241 |
|  | R | GCA TAC CAC ATA GTT TGT TTA GGT G | NH1125  NH1067 | 1266-1290 |
| **3** | F | GAG ATG AAG CTA CTG CAC AAA GTA GGG | NH1125 | 821-848 |
|  | R | CCA TAG GCA TTC ATA AAC AAT CCT GC | NH1125 | 1869-1894 |
| **4** | F | GCT CAA TAG TTA AGA AGG AGC TAA TCC G | NH1125  NH1067 | 1055-1082 |
|  | R | GCA TTA CTT GCC CTG AAC CAT AGG | NH1125  NH1067 | 1888-1911 |
| **5** | F | GGCTCTTAGCAAAGTCAAGTTRAATGATAC | NH1125 | 1140-1169 |
|  | R | ACC ATA GGC ATT CAT AAA YAA TCC TGC | NH1125 | 1867-1893 |
| **6** | F | GGA GGG CAA ACA ATG TC | NH1125  NH1067 | 1656-1674 |
|  | R | GGG TGA GAT CTT CTT TGA AGC | NH1125  NH1067 | 2604-2624 |
| **7** | F | CCC GAT AAC ATC TGG CAC CAA | NH1125  NH1067 | 2509-2529 |
|  | R | CCC ATA TTG TTA GTG ATG CAG GAT CAT CAT C | NH1125  NH1067 | 3338-3368 |
| **8** | F | CTC ACT CAG CAA TCA ACA ACA TC | NH1125  NH1067 | 3082-3108 |
|  | R | CTG TGA TAA CTA ATA CTA ATC CTG CAT AGG G | NH1125  NH1067 | 3846-3876 |
| **9** | F | CAA TCC ATT GAA TCA ACT GCC AGA C | NH1125 | 3122-3146 |
|  | R | GAG ATT AGA GTT AAG ATC ATA TGT ATT AGT GTA A | NH1125 | 4355-4388 |
| **10** | F | GAT GTA ACT ACA CCT TGT GAA ATC AAA GC | NH1125 | 3573-3601 |
|  | R | CCA TGA CTC TGT GAG AAG ATT GG | NH1125 | 4554-4576 |
| **11** | F | GATCTTACCATGAAGACATTCAACCC | NH1067 | 3650-3676 |
|  | R | GAT TTG ATA CAT CTG TCC TTG TTC AAG AG | NH1067 | 4461-4479 |
| **12** | F | CCA GCC ACT ATC TGC TAG ACC | NH1125  NH1067 | 4164-4184 |
|  | R | GCT AAC CCT TTC TGG TGG GAC | NH1125  NH1067 | 4969-4989 |
| **13** | F | CCGCGGGTTCTGGCAATGATAATCTCAAC | NH1125 | 4819-4844 |
|  | R | GTT CTT GCT TGA TAG ATC ACG GTT C | NH1125 | 5614-5638 |
| **14** | F | CCGCGGGTTCTGGCAATGATAATCTCAAC | NH1125  NH1067 | 4819-4844 |
|  | R | GGG GCC CCG CGG CCG CGC ATT AAT AGC AAG AGT TAG GAA G | NH1125  NH1067 | 5698-5708 |
| **15** | F | CAC AAA GTT ACA CTA ACA ACT GTC AC | NH1125 | 4886-4911 |
|  | R | CCT ACA CCT AAC AAG AAG CCC | NH1125 | 6079-6099 |
| **16** | F | CAC AAA GTT ACA CTA ACA ACG GTT ACA G | NH1125  NH1067 | 4886-4913 |
|  | R | GTT CTT GCT TGA TAG ATC ACG GTT C | NH1125  NH1067 | 5614-5638 |
| **17** | F | CAC CAG CAC CTC ACA ATC CAC | NH1125  NH1067 | 5418-5438 |
|  | R | GTT GTT ACA CCT GCA TTG ACA CTG | NH1125  NH1067 | 6376-6400 |
| **18** | F | CCC ACA GCA TCC GAG CCC TC | NH1125 | 5530-5549 |
|  | R | GCA ATA CAA CAG CAA ACC AAT AGC | NH1125 | 7290-7321 |
| **19** | F | CAC CTT GAA GGA GAA GTG AAC AAG ATC | NH1125  NH1067 | 6140-6166 |
|  | R | CAT CAG AAG GAA ACA CTA GAG GGT C | NH1125  NH1067 | 7100-7125 |
| **20** | F | GGA GTA GAT ACT GTG TCA GTG GGC | NH1125  NH1067 | 7001-7024 |
|  | R | CGC TGG TGG TTA TCC AAA TGG TTA TGG | NH1125  NH1067 | 8290-8316 |
| **21** | F | GAT ACT GTG TCA GTG GGC AAC AC | NH1125 | 7007-7029 |
|  | R | CCT ATG TAA CTC TCT AGC ACT CCA AC | NH1125 | 7846-7871 |
| **22** | F | GAC CCT CTA GTG TTT CCT TCT GAT GAG | NH1125 | 7068-7094 |
|  | R | CAC TTG AGA TCA ATA TAG ATG ATA TAC TAC AAG | NH1125 | 8178-8210 |
| **23** | F | GCA GAC GTG CTG AAG AAG AC | NH1125 | 8077-8096 |
|  | R | GTA TAT GAT TGA TTG CTT TCC ACA GCC G | NH1125 | 8993-9020 |
| **24** | F | GCA GAC GTG CTG AAG AAG AC | NH1067 | 8077-8096 |
|  | R | CGC TGG TGG TTA TCC AAA TGG TTA TGG | NH1067 | 8290-8316 |
| **25** | F | GTG AAA GCA TGA TTG CTA CAT TCA ATC | NH1125 | 8241-8267 |
|  | R | GAG TCC TTT ATG ATA AAC GAT ACA ACC | NH1125 | 9256-9282 |
| **26** | F | GTA GAC AAA GCC CAC TAC TAG AGC | NH1067 | 8657-8680 |
|  | R | CCA TGA TGG AGG ATG TTG CAT TGA AC | NH1067 | 9092-9117 |
| **27** | F | GCA TGT CCT CGT CTG AAC AAA TTG C | NH1067 | 8795-8819 |
|  | R | CCA TTG CTT GTC TTT CAT CAA CCA TTG G | NH1067 | 9802-9829 |
| **28** | F | GAC AAA GCC CAC TAC TAG AGC | NH1125 | 8660-8680 |
|  | R | GTG TGA CAT ACT CTT GAT AGT AGG TCC | NH1125 | 9627-9653 |
| **29** | F | GGA AAG ACA TCA GCC TTA GCA GAT TAA ATG | NH1125  NH1067 | 9323-9352 |
|  | R | CTC TCA AGT AAT ACT CTA GTA CTC TTC TTG | NH1125  NH1067 | 10244-10273 |
| **30** | F | CAG GAT TGC GGT TCT ATC GTG AG | NH1067 | 10058-10080 |
|  | R | GCA TGA TTT CGG AGT TGC AGA GC | NH1067 | 11243-11266 |
| **31** | F | GAC AAA GCC ATT TCA CCT CC | NH1125  NH1067 | 10123-10142 |
|  | R | CTC CTG TGT TAA GCT ACC TAT AGA TTC | NH1125  NH1067 | 11223-11250 |
| **32** | F | GAC AAA GCC ATT TCA CCT CC | NH1125  NH1067 | 10123-10142 |
|  | R | CAG TCT ATC ATC TGG AAG ATC CTG G | NH1125  NH1067 | 11592-11616 |
| **33** | F | CAA TTC TTC CCT GAG AGT TTG ACA AG | NH1067 | 10463-10489 |
|  | R | GTC TGA CCC TCT ATA AGT CTA ACT GG | NH1067 | 10964-10990 |
| **34** | F | GGA GTG TAC TAT CCA GCC AGT ATC | NH1125  NH1067 | 11137-11161 |
|  | R | CAA CTC TTA ATC CAT GAG GGT AAG TTG G | NH1125  NH1067 | 11854-11881 |
| **35** | F | GGA GAA CTC CAG ACT TCC TTA CAG | NH1125  NH1067 | 11504-11527 |
|  | R | GTT CCA CAA CCG ACA TCA GGC | NH1125  NH1067 | 12689-12709 |
| **36** | F | GGG TAG GTT CAT CTA CGC AGG AG | NH1125  NH1067 | 12293-12315 |
|  | R | CTG GGG TTG GGT GAT ATA GTT TG | NH1125  NH1067 | 13617-13639 |
| **37** | F | CCG TAT GCC CTT GGG TTG | NH1125  NH1067 | 13370-13387 |
|  | R | GGC TGT AAC AGG TAA TTC AGC ATC GC | NH1125  NH1067 | 14312-14337 |
| **38** | F | CCA GTT GTA TAG CAT TCA TAG GTG AAG G | NH1125  NH1067 | 14045-14066 |
|  | R | GTT GCT GAA TAC TTC ATT ACG TCC AGC | NH1125  NH1067 | 14771-14796 |
| **39** | F | AACCTATTAGCATCTTTGTCTGCGATGCTG | NH1125  NH1067 | 14291-14320 |
|  | R | CTAATGTCTCGTTGTGTTGTAAATGCACATG | NH1125  NH1067 | 15173-15203 |
| **40 ^b^** | F | CAGGTAGTGTACTATACAACCTTCCCAAT | NH1125  NH1067 | 14971-14999 |
|  | F | AAAATTATCTATTTTGGTCTTAAGGGGTTAAAT | NH1125  NH1067 | 15116-15148 |
|  | R | GCACCCCATTTCTGATTGAAAAGG | NH1125  NH1067 | 84-107 |
|  | R | GCCGTTTAATTTAATTGTATGTATTGCTGC | NH1125  NH1067 | 228-257 |
|  | R | CAGGGCACACTTCACTGCTTGTTA | NH1125  NH1067 | 277-300 |

1. Virus specific primers were designed based on acquired sequence data
2. Combination of these primers were used to amplify and sequence the ends of the genome after ligation with T4 RNA ligase
